# Effect of Formulation Variables on Preparation of Celecoxib Loaded Polylactide-Co-Glycolide Nanoparticles

**DOI:** 10.1371/journal.pone.0113558

**Published:** 2014-12-12

**Authors:** Dustin L. Cooper, Sam Harirforoosh

**Affiliations:** Department of Pharmaceutical Sciences, Gatton College of Pharmacy, East Tennessee State University, Johnson City, Tennessee, United States of America; RMIT University, Australia

## Abstract

Polymer based nanoparticle formulations have been shown to increase drug bioavailability and/or reduce drug adverse effects. Nonsteroidal anti-inflammatory drugs (e.g. celecoxib) reduce prostaglandin synthesis and cause side effects such as gastrointestinal and renal complications. The aim of this study was to formulate celecoxib entrapped poly lactide-co-glycolide based nanoparticles through a solvent evaporation process using didodecyldimethylammonium bromide or poly vinyl alcohol as stabilizer. Nanoparticles were characterized for zeta potential, particle size, entrapment efficiency, and morphology. Effects of stabilizer concentration (0.1, 0.25, 0.5, and 1% w/v), drug amount (5, 10, 15, and 20 mg), and emulsifier (lecithin) on nanoparticle characterization were examined for formula optimization. The use of 0.1, 0.25, and 0.5% w/v didodecyldimethylammonium bromide resulted in a more than 5-fold increase in zeta potential and a more than 1.5-fold increase in entrapment efficiency with a reduction in particle size over 35%, when compared to stabilizer free formulation. Nanoparticle formulations were also highly influenced by emulsifier and drug amount. Using 0.25% w/v didodecyldimethylammonium bromide NP formulations, peak zeta potential was achieved using 15 mg celecoxib with emulsifier (17.15±0.36 mV) and 20 mg celecoxib without emulsifier (25.00±0.18 mV). Peak NP size reduction and entrapment efficiency was achieved using 5 mg celecoxib formulations with (70.87±1.24 nm and 95.55±0.66%, respectively) and without (92.97±0.51 nm and 95.93±0.27%, respectively) emulsifier. In conclusion, formulations using 5 mg celecoxib with 0.25% w/v didodecyldimethylammonium bromide concentrations produced nanoparticles exhibiting enhanced size reduction and entrapment efficiency. Furthermore, emulsifier free formulations demonstrated improved zeta potential when compared to formulations containing emulsifier (p<0.01). Therefore, our results suggest the use of emulsifier free 5 mg celecoxib drug formulations containing 0.25% w/v didodecyldimethylammonium bromide for production of polymeric NPs that demonstrate enhanced zeta potential, small particle size, and high entrapment efficiency.

## Introduction

Nonsteroidal anti-inflammatory drugs (NSAIDs) are well established for the treatment of pain and inflammation. They function by acting on the cyclo-oxygenase (COX) family of enzymes and inhibiting the conversion of arachidonic acid to prostaglandins and thromboxanes [1], [2]. The COX enzyme exists as at least two different isozymes, COX-1 and COX-2. The COX-1 enzyme is constitutively expressed in most tissue and functions to regulate hemodynamics and maintain gut integrity. COX-2 is an inducible enzyme found primarily at sites of inflammation that mediates fever and pain [3]–[5]. COX-2 has been found to be constitutively expressed in certain tissue such as the kidneys, the reproductive tract, and gastric mucosa [6]–[9]. Traditionally, NSAIDs function by inhibiting both COX-1 and COX-2 isozymes and provide analgesic and anti-inflammatory benefits. These benefits are thought to arise primarily from the inhibition of COX-2, while the adverse effects (e.g. ulceration) were thought to occur from over inhibition of COX-1 [10]–[12]. As a result, COX-2-selective inhibitors (COXIBs) were developed to provide analgesic and anti-inflammatory benefits, while minimizing the gastrointestinal adverse side effects associated with traditional NSAID use [10], [13].

Celecoxib (CEL) is a COXIB used in the treatment of pain and inflammation [11], [12], [14]. Evidence suggests that CEL use effectively reduces clinical gastrointestinal events in comparison to other NSAIDs, making it one of the most commonly prescribed COX-2 specific inhibitors [15]–[17]. Despite the general safety of CEL in regard to gastrointestinal tolerability, its use has been associated with the development of several adverse side effects including cardiovascular events, and renal toxicity [17], [18]. Many CEL delivery systems have been developed to reduce CEL associated side effects [19]–[22]. Studies utilizing nanoparticle (NP) formulations have shown promising results in overcoming high dose oral administration of CEL [21], [23]–[25]. One study showed enhanced drug retention at the site of action following intra-articular injection of small lipid nanoparticle formulated CEL in the treatment of joint pain [26]. Another study showed enhanced anti-inflammatory effects of CEL utilizing NP formulated transdermal drug delivery [27]. A third study showed enhanced inhibition of tumor growth with a reduction in side effects using hydroxyapatite-chitosan nanocomposited CEL in the treatment of colon cancer [28].

Polymer based NPs are commonly used to improve drug bioavailability and/or reduce drug associated side effects [29]. Poly lactide-co-glycolide (PLGA) is a polymer that has been commercialized for a variety of drug delivery systems and is frequently used in the design of biocompatible NPs [30]. PLGA is approved by the Food and Drug Administration as a biodegradable polymer that degrades to the nontoxic tricarboxylic acid cycle intermediates, lactic acid and glycolic acid [30]–[32]. Use of PLGA based NPs for enhanced delivery of CEL has been met with a variety of results [19], [20]. However, known NP stabilizers such as didodecyldimethylammonium bromide (DMAB) and poly vinyl alcohol (PVA) have yet to be used in the development of CEL loaded PLGA-NPs.

Previous studies have shown effective use of DMAB and PVA for formulation of small, highly entrapped NPs [33], [34]. The aim of this study was to characterize and optimize CEL loaded PLGA-NPs by examining the influence of varying DMAB and PVA concentrations on NP characterstics. The effect of drug amount and emulsifier (lecithin) on zeta potential, particle size, entrapment efficiency, morphology, and stability was also examined.

## Materials and Methods

### Materials

DMAB, PVA (MW 89,000–98,000 Da, 99.9+% hydrolyzed), PLGA (50∶50 copolymer compositions; MW 30,000–60,000 Da), and lecithin (99% phosphatidylcholine) were purchased from Sigma-Aldrich (St. Louis, MO, USA). CEL base powder was obtained from Biovision Incorporated (Milpitus, CA, USA). Acetone, ethyl acetate, and high-performance liquid chromatography (HPLC)-grade water were purchased from Fischer Scientific Laboratory (Fair Lawn, NJ, USA).

### Preparation of CEL loaded PLGA-NPs

NP formulations were carried out using a previously described solvent evaporation technique [33], [35]. CEL-loaded NPs were formulated by dissolving 20 mg of CEL and 50 mg PLGA into 3 mL of ethyl acetate. The solution was stirred for 30 minutes at 750 rpm. Afterwards, 30 mg of lecithin was added to the organic solution followed by addition of 500 µL acetone as co-solvent. A varying range of DMAB or PVA concentrations (0.1%, 0.25%, 0.5%, and 1% w/v) was dissolved in 6 mL of HPLC grade water. Organic phase was then added to aqueous phase in a drop wise manner under moderate stirring followed by sonication for 5 minutes at 20 KHz. After sonication, solutions were stirred at 750 rpm for 1 hour to evaporate organic phase. Emulsions were then centrifuged at 12,000 rpm followed by separation of supernatant from precipitants. Additional NP formulations for optimization studies were carried out with 0.25% w/v DMAB concentration. Using the previously described process, emulsifier free CEL loaded PLGA-NPs were formulated with the exclusion of lecithin; while NP preparation for observing the effects of drug amount was carried out using various amounts of CEL (5, 10, 15, and 20 mg).

### Particle size and zeta potential of CEL-loaded NPs

Intensity weighted mean particle size (diameter) was measured in triplicate by dynamic light scattering using a NICOMP particle sizer (Particle Sizing Systems, Port Richy, FL, USA). Zeta potential was estimated on the basis of electrophoretic mobility under an electrical field.

### Drug entrapment efficiency

To measure drug entrapment efficiency, 100 µL NP formulation was added to 300 µL acetonitrile and vortex mixed for 30 seconds. Afterwhich, 100 µL of drug loaded NP solution was analyzed under ultraviolet–visible spectroscopy (Eppendorf Biophotometer, Hauppauge, NY, USA) at 260 nm using empty NP solutions as blank. A standard calibration curve (50,000–2,000,000 ng/mL) was constructed using titrated dilutions of CEL stock solution dissolved in acetonitrile. Drug entrapment efficiency was calculated using the following equation:

Entrapment efficiency (%)  =  (Amount of CEL entrapped within nanoparticles/Total amount of CEL used for formulation) ×100

### Morphology

Transmission electron microscopy (TEM) (Tecnai Philips Transmission Electron Microscope; FEI, Hillsboro, Oregon, USA) was used for evaluation of CEL loaded PLGA-NP shape and surface morphology. NP emulsions were vortex mixed and 2 µL aliquots were placed on a 200 mesh copper grid covered with Formvar film (Electron Microscopy Sciences, Hatfield, Pennsylvania). Samples were air dried for 1 hour then examined at 80 kV.

### Stability of CEL loaded PLGA-NPs

CEL loaded PLGA-NP emulsions (5 mL) formulated at various drug amounts (5, 10, 15, and 20 mg) with or without emulsifier (0.25% w/v DMAB) were stored at 4°C for a period of 16 weeks. After 16 weeks samples were removed from storage and analyzed for particle size, zeta potential, and drug entrapment efficiency. Particle characteristics were evaluated as previously described.

### Data analysis

All experiments were performed in triplicate. NP characteristic data is represented as mean ± standard deviation (SD). A Student's t-test was used for comparison of two groups.

## Results and Discussion

### Effect of stabilizer concentration on NP characteristics

**Figure 1:**
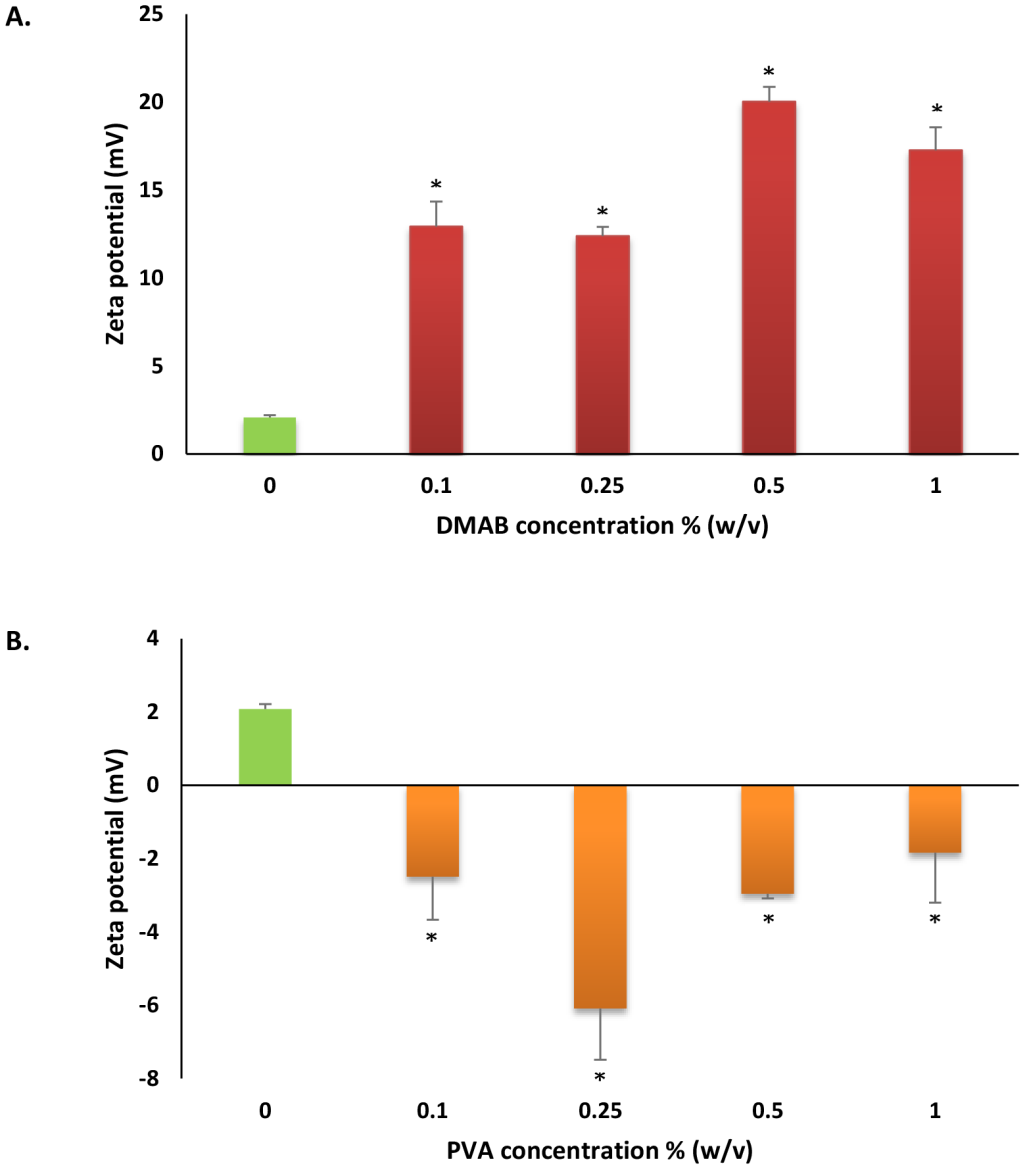
Zeta potential measurements of A) DMAB and B) PVA formulated NPs of celecoxib. Values are expressed as mean ± SD, n = 3. ^*^p<0.05, significantly different from plain formulation.

CEL encapsulated PLGA-NPs were developed using lecithin as an emulsifier with DMAB or PVA (Table 1). The use of DMAB or PVA resulted in formation of CEL loaded PLGA-NPs with surface characteristics that displayed positive and negative charges, respectively (Fig. 1). Because of the cationic properties of DMAB [36]–[38], NPs formulated with inclusion of DMAB showed highly positive surface charges (Fig. 1A). DMAB formulated CEL loaded NPs reached a peak zeta potential of 20.03±0.84 mV at 0.5% w/v concentration. The anionic characteristics of PVA led to the formation of NPs with slightly negative surface charges (Fig. 1B). PVA formulated NPs reached a peak zeta potential of −6.09±1.39 mV with 0.25% w/v concentration.

**Table 1:** NP formulation with varying concentrations of DMAB or PVA.

Formulation Number	Ingredients										
	Ethyl acetate (mL)	Water (mL)	DMAB (% w/v)	PVA (% w/v)	PLGA (mg)	Acetone (µL)		Celecoxib(mg)		Lecithin (mg)	
1*	3	6	-	-	50		500		20		30
2	3	6	0.1	-	50		500		20		30
3	3	6	0.25	-	50		500		20		30
4	3	6	0.5	-	50		500		20		30
5	3	6	1	-	50		500		20		30
6	3	6	-	0.1	50		500		20		30
7	3	6	-	0.25	50		500		20		30
8	3	6	-	0.5	50		500		20		30
9	3	6	-	1	50		500		20		30

^*^Stabilizer free (plain) formulation.

When comparing zeta potential as a measure of stability, all CEL-NP formulations containing DMAB or PVA showed significant alterations in NP system stability compared to stabilizer free formulations (plain formulation) (Fig. 1A and Fig. 1B). These results are indicative of altered NP characteristics as a result of adsorption or inclusion of DMAB and PVA onto or within the NP polymer shell. The inclusion of cationic and anionic DMAB (Fig. 1A) or PVA (Fig. 1B) on NP surfaces can effectively alter overall NP charge, in turn, effecting overall system stability [38]–[40].

**Figure 2:**
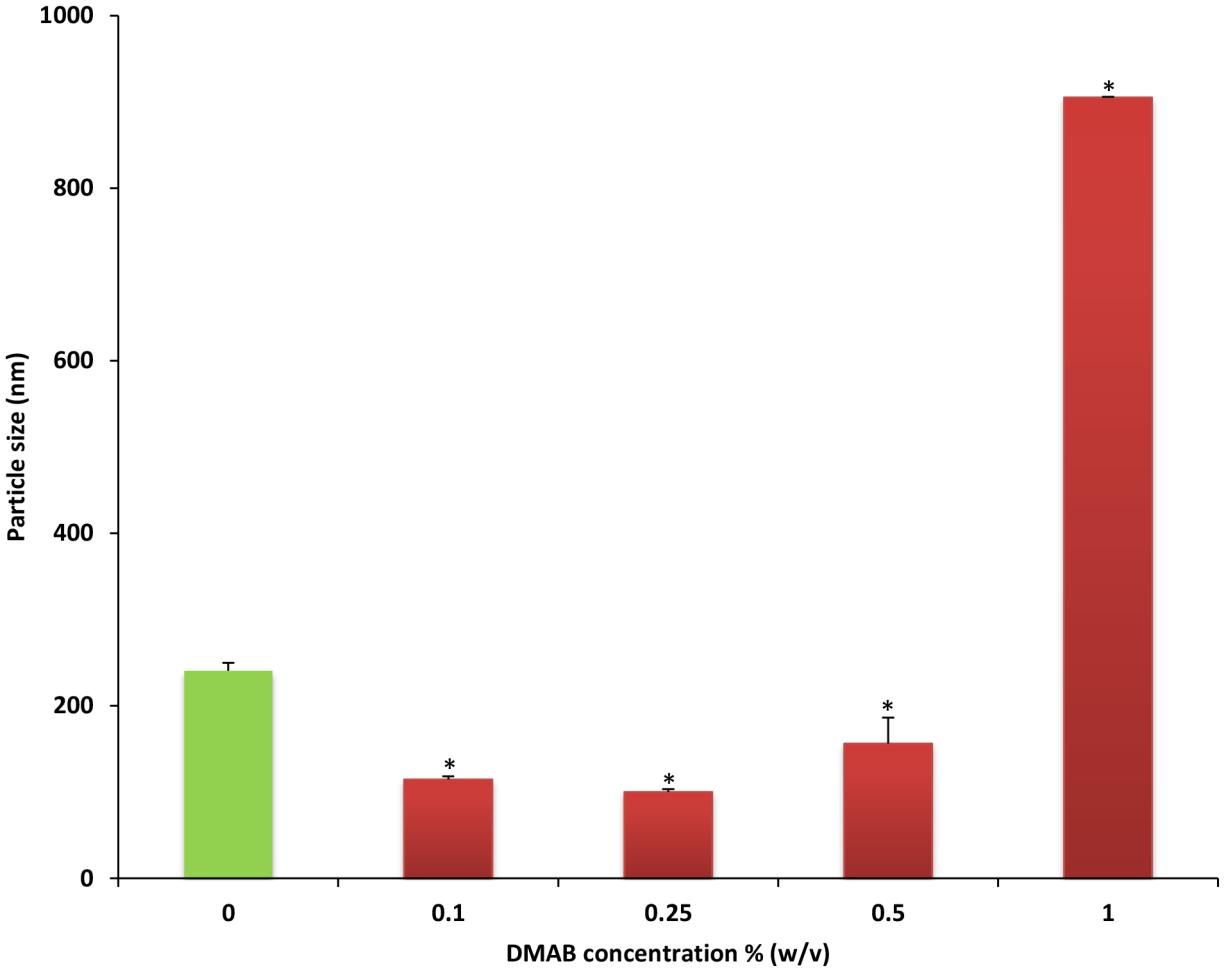
Particle size analysis of increasing concentrations of DMAB compared to formulation without stabilizer (plain formulation). Values are expressed as mean ± SD, n = 3. ^*^p<0.05, significantly different from plain formulation.

In comparison to plain formulation, a significant reduction in particle size was seen in formulations incorporating 0.1%, 0.25%, and 0.5% DMAB (Fig. 2). Particle size was significantly increased in 1% DMAB concentrations when compared to plain formulation. The largest reduction in particle size was achieved using 0.25% DMAB concentration (99.97±3.27 nm) (p<0.01). High concentrations of DMAB have been shown to increase system viscosity, resulting in a direct increase in particle size [41], which may explain the significant rise in particle size noticed with CEL-NPs formulated using larger amounts of stabilizer. Furthermore, DMAB can act as a solubilizing agent for known hydrophobic compounds [35]. It is possible that lower DMAB concentrations may act to effectively reduce drug crystallization, further reducing NP size, which may explain NP size reductions seen in our study with lower DMAB concentrations (Fig. 2). Conversely, a significant increase in CEL solubility brought forth by higher DMAB content could also function to increase NP drug loading capacity and increase particle size by means of expanding NP drug content within the polymer shell.

Formulations using 0.1% w/v PVA did not demonstrate any significant difference in particle size when compared to plain formulation (p>0.77). Particle size measurements of 0.25%, 0.5% and 1% PVA formulations were not detectable by our NICOMP particle sizer due to reduced entrapment efficiency and total drug concentrations in PVA based NP solution.

The amount entrapped (1.99±0.01 mg) and entrapment efficiency (9.94±0.01%) of CEL in formulations without stabilizer were compared to DMAB and PVA based formulations (Table 2). All stabilizer based formulations demonstrated significant changes in entrapment efficiency when compared to plain formulation (Table 2) (P<0.01). All DMAB formulations and 0.1% PVA formulation exhibited significant increases in the level of CEL entrapment with a maximum percent entrapment of 61.07±0.06% reached with 1% DMAB formulation. All PVA concentrations above 0.1% w/v underwent a significant reduction in drug entrapment (Table 2). The reduction in drug entrapment can be explained by elucidation of PVA properties. PVA is a highly hydrophilic stabilizer, which can result in reduced NP stability in aqueous solutions [42]. As PVA concentrations increase, the hydrophilic nature of the NP system increases. The increased inclusion of PVA into the NP polymer shell could increase hydrophilic properties leading to NP solubilization in the aqueous medium following organic phase evaporation. The increased hydrophilic properties of PVA-NP systems could reduce entrapment efficiency leading to an increased loss of drug in solution precipitant following centrifugation.

**Table 2:** Effects of stabilizer concentrations on celecoxib entrapment.

Stabilizer	Conc. (% w/v)	AE (mg)	EE (%)
Stabilizer free	0	1.99±0.01	9.94±0.01
DMAB	0.1	3.78±0.01	18.85±0.07*
	0.25	9.94±0.08	49.70±0.38*
	0.5	6.16±0.01	30.84±0.04*
	1	12.22±0.01	61.07±0.06*
PVA	0.1	9.23±0.03	46.19±0.16*
	0.25	0.11±0.02	0.56±0.03*
	0.5	0.06±0.01	0.33±0.02*
	1	0.66±0.03	3.28±0.14*

All values reported as mean ± SD (n = 3). Amount entrapped (AE) per 20 mg celecoxib. EE is the entrapment efficiency.

^*^P<0.01 compared to plain formulation.

In this study, DMAB was shown to effectively increase zeta potential, reduce particle size, and facilitate drug entrapment when compared to PVA based formulations. As such, DMAB based NP morphology was visualized and confirmed under transmission electron microscopy (TEM) (S1 Figure) with further variable analysis carried out using DMAB formulations.

### Analysis of NP characteristics in absence of emulsifier

**Figure 3:**
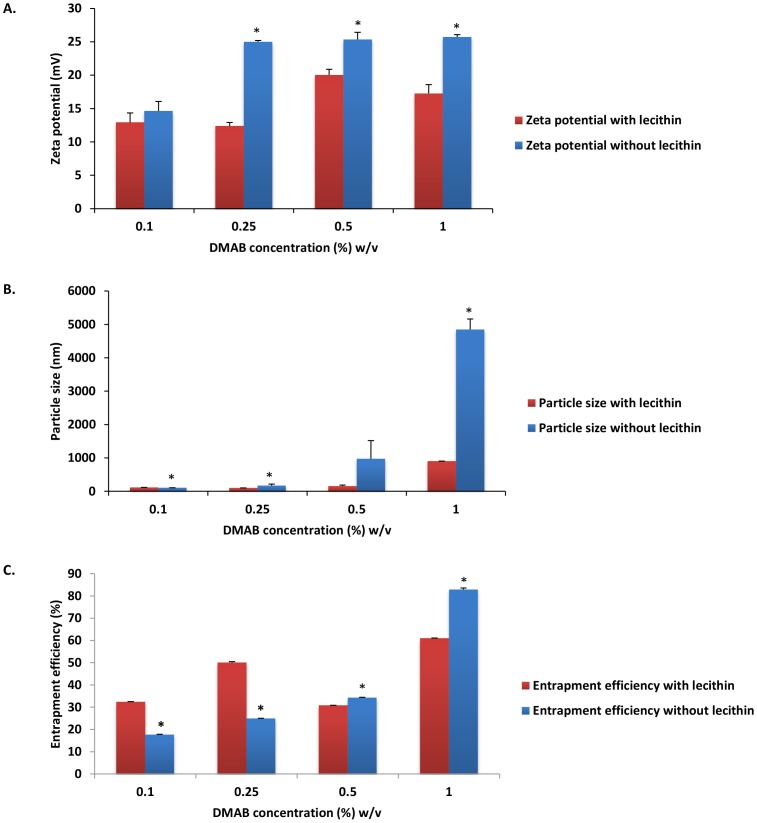
Nanoparticle characteristic comparison of A) zeta potential, B) particle size, and C) entrapment efficiency of initial emulsifier based DMAB formulations with emulsifier free DMAB formulations. Values are expressed as mean ± SD, n = 3. ^*^p<0.05, significantly different from initial formulations.

To analyze the effect of emulsifier on CEL loaded NP characteristics, formulations containing 0.1%, 0.25%, 0.5%, and 1% w/v DMAB without lecithin were developed, characterized, and compared to previously observed characteristics of NP formulations with lecithin (Fig. 3). NP visual identification of emulsifier free formulations was performed via TEM analysis (S2 Figure). When compared to emulsifier based formulations, absence of emulsifier resulted in a significant increase of zeta potential in formulations using 0.25%, 0.5%, and 1% stabilizer concentrations (Fig. 3A) (P<0.01). These findings indicate that the use of an emulsifier may function to reduce overall particle repulsion and system stability. The cationic property of DMAB has become increasingly popular for development of positively charged NPs [43]. In emulsifier free formulations, we found that rising zeta potential was associated with increased DMAB concentration. These results can be indicative of enhanced DMAB inclusion into the NP polymer shell. Furthermore, lecithin contains low concentrations of phosphatidic acid. The presence of phosphatidic acid can impart negatively charged, anionic characteristics during inclusion into NP formulations [44]. As such, the anionic properties of lecithin can act to reduce polymer surface charge and effectively mask the cationic charge associated with DMAB inclusion, which would explain the findings of reduced particle charge seen in emulsifier based NP formulations.

In emulsifier free formulations, particle size increased with increasing stabilizer concentrations, with peak particle size reaching micron levels at 0.5% and 1% DMAB concentration (972.93±547.71 nm and 4849.77±313.75 nm, respectively) (Fig. 3B). These results indicate that lecithin effectively reduces interfacial tension between organic and aqueous phases. In solvent evaporation processes, when organic phase is added to aqueous phase in a drop wise manner, the resultant organic droplets are stabilized by polymers formed at solute interfaces [44]. The type of polymer, surfactant, or emulsifier used can act to alter interfacial tensions between the organic droplets and the aqueous solution. After placement of organic phase into aqueous phase, interfacial spreading occurs as a result of diffusion between solvents, providing energy for NP formation [44]. NP size is dependent on diffusion rate which is dependent on changes in the interfacial tension between organic and aqueous phases. Lower interfacial tension equates to smaller NP size properties [44]–[47]. The addition of compounds such as lecithin act to effectively change interfacial tension which can alter particle size and NP formation [47], [48]. Lecithin favors a higher organic phase to aqueous phase interface [45] that, when added to organic solvents such as ethyl acetate, could function to alter the rate of solvent diffusion and reduce particle size.

Peak drug entrapment was seen at DMAB concentrations of 1% for formulations without emulsifier (82.91±0.67%) (Fig. 3C). In relations to 1% DMAB formulation carried out with emulsifier, these results equate to an almost 22% increase in NP drug loading (P<0.01). In theory, inclusion of lecithin could act to offset surface tension allowing for fast organic phase diffusion into the aqueous phase [49]. The alteration in interfacial tension could also function to reduce barrier transport of drug outside of the organic phase during solvent diffusion. Inclusion of lecithin into the polymeric shell with increasing concentrations of DMAB resulted in a net reduction in drug entrapment compared to its emulsifier free counterpart. Much like lecithin, DMAB can form micelle aggregates that function through hydrophobic interactions of DMAB with the hydrophobic core of the NP [33]. The interactions of the hydrophobic portion of the stabilizer can function to solubilize the hydrophobic drug entrapped within the NP core [35]. It is possible that as concentrations of both DMAB and lecithin increased in formulations, the net rise in hydrophobic interaction resulted in increased NP and CEL solubility leading to drug leakage and reduced drug entrapment. Similar results were obtained by Thakkar *et al.* when using Span-85 as an emulsifying agent during the development of CEL microspheres [50]. In the study, it was found that formulations using high concentrations of emulsifier (5% w/w) and stabilizer (2% w/w) resulted in enhanced CEL solubility and dissolution, which led to a reduction in both particle size and drug entrapment efficiency.

Previously, our lab completed formulation of diclofenac (a non-selective NSAID) loaded PLGA-NPs using DMAB and PVA [33]. With no change in drug amount (45 mg) or use of emulsifier, diclofenac loaded PLGA-NPs with DMAB or PVA exhibited negative surface charges and a peak entrapment efficiency as high as 80.21±1.21%. The negative NP surface charge associated with diclofenac NP formulations using DMAB contrast with the highly positive surface charge of DMAB formulated CEL loaded NPs found in this study. When using diclofenac, PVA formulated NPs showed smaller negative surface charges, similar to the negative surface charge characteristics associated with our PVA formulated CEL-NP formulation. In physiological conditions, diclofenac is a negatively charged molecule which may play a role in the development of negatively charged NPs during formulation with DMAB [51]. Conversely, at physiological pH, CEL presents as a neutrally charged molecule [52] that, when formulated with cationic DMAB, resulted in formation of positively charged NPs. Particle size analysis showed a similar pattern when comparing CEL formulation results with that of diclofenac. Diclofenac NP formulation showed a maximum increase of NP size (189.9±4.9 nm) using 1% w/v DMAB. Similarly, results of our CEL formulation study demonstrated maximum NP size with 1% w/v DMAB concentration (Fig. 3B). Measurements of entrapment efficiency showed opposite effects. For diclofenac NPs, a linear reduction in total entrapment efficiency was seen with regard to increasing DMAB concentrations with the lowest amount of diclofenac entrapment occurring with 1% w/v DMAB. Conversely, maximum CEL loading seen within this study was observed at 1% DMAB, which when compared to the highly polarizable diclofenac [53],

the theory that higher concentrations of DMAB may increase solubility of lipophilic drugs such as CEL, in turn leading to increases in particle size and drug entrapment [41].

### Effect of drug amount on NP characteristics

**Figure 4:**
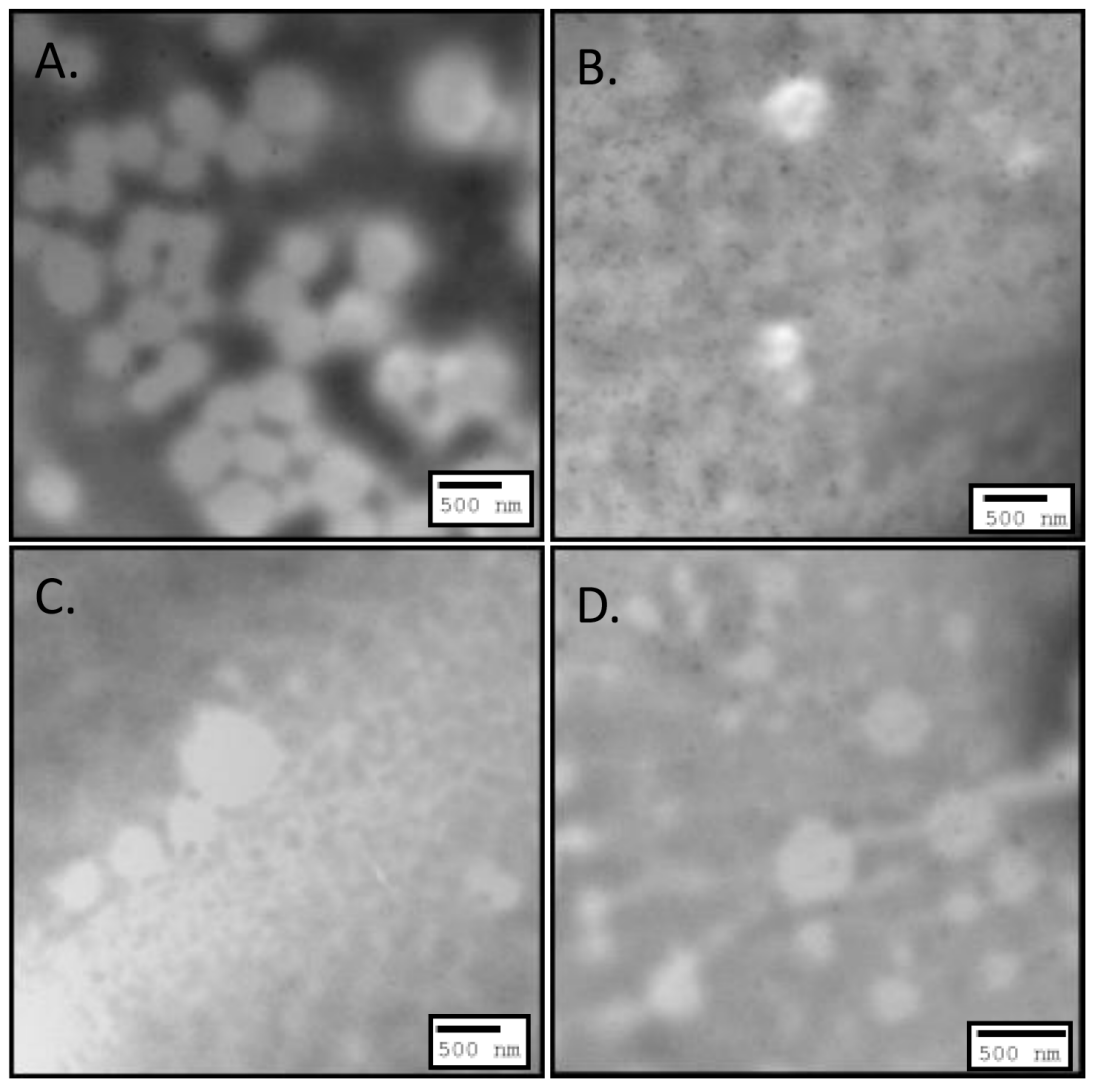
TEM images illustrating morphology of 0.25% w/v DMAB NP formulations with emulsifier at A) 5 mg drug amount, B) 10 mg drug amount, C) 15 mg drug amount, and D) 20 mg drug amount.

**Figure 5:**
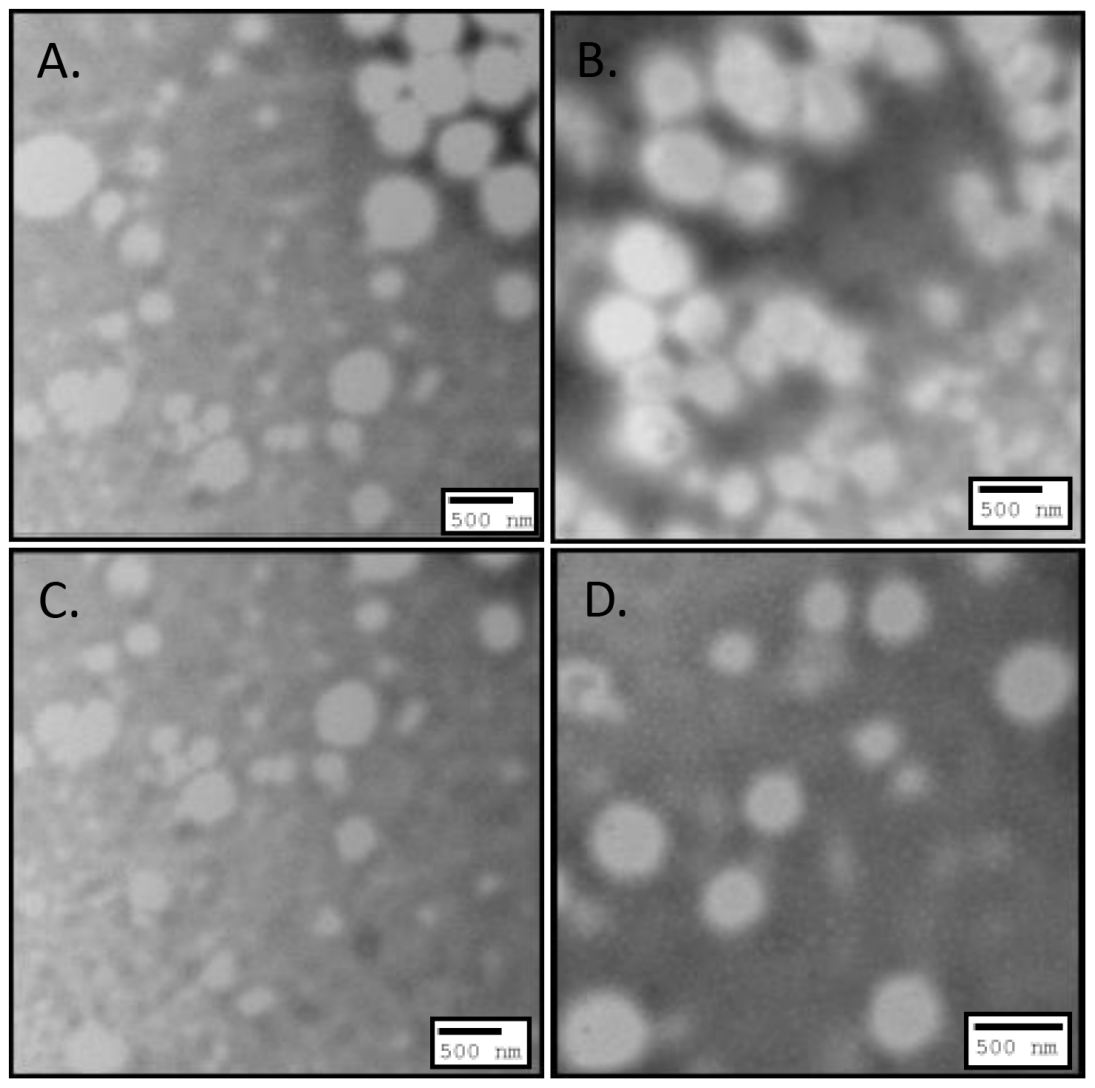
TEM images illustrating morphology of 0.25% w/v DMAB NP formulations without emulsifier at A) 5 mg drug amount, B) 10 mg drug amount, C) 15 mg drug amount, and D) 20 mg drug amount.

**Figure 6:**
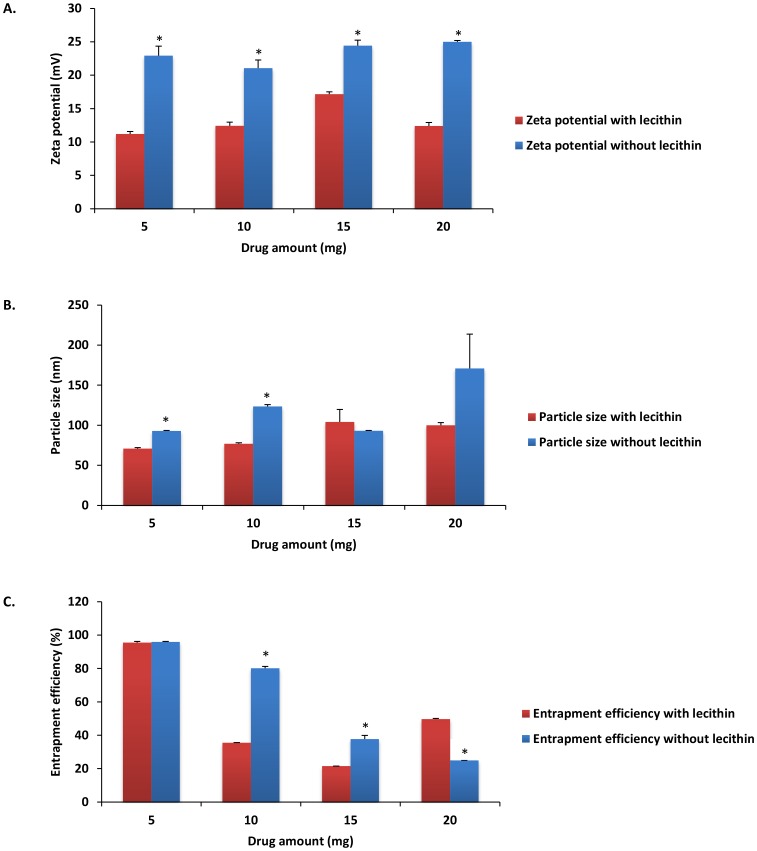
The effect of varying drug amounts on A) zeta potential, B) NP size, and C) entrapment efficiency. Values are expressed as mean ± SD, n = 3. ^*^p<0.05, significantly different from formulations with emulsifier.

To study the effect of drug amount on NP characteristics, formulations consisting of 0.25% DMAB concentrations were chosen based on their sufficient size and general representation of drug entrapment and zeta potential. In conjunction with previously formulated NP systems using 20 mg CEL, new NPs with or without emulsifier were formulated with increasing amounts (5, 10, and 15 mg) of CEL (Table 3). Morphological characterization of NPs formulated with (Fig. 4) and without (Fig. 5) emulsifier at various drug amounts showed spherical shape and size similar to what was noticed in previous formulation studies [23], [24], [54]–[58]. All NP formulations without emulsifier displayed significantly higher zeta potential compared to formulations with emulsifier (Fig. 6A) (p<0.01). Maximum zeta potential was reached in formulations of 20 mg CEL without emulsifier (25.00±0.18 mV). Formulations with emulsifier reached peak zeta potential using 15 mg CEL (17.15±0.36 mV). These results further indicate that use of emulsifiers such as lecithin, can function to mask surface charge of the incorporated stabilizer thereby reducing overall cationic charge associated with DMAB formulated NPs [44].

**Table 3:** Preparation method for NP formulations with differing drug amounts.

Formulation Number	Ingredients						
	Ethyl acetate (mL)	Water (mL)	DMAB (% w/v)	PLGA (mg)	Acetone (µL)	Celecoxib (mg)	Lecithin (mg)
1	3	6	0.25	50	500	20	30
2	3	6	0.25	50	500	15	30
3	3	6	0.25	50	500	10	30
4	3	6	0.25	50	500	5	30
5	3	6	0.25	50	500	20	-
6	3	6	0.25	50	500	15	-
7	3	6	0.25	50	500	10	-
8	3	6	0.25	50	500	5	-

Peak size reduction and entrapment efficiency for formulations with (70.87±1.24 nm and 95.55±0.66%, respectively) and without (92.97±0.53 nm and 95.93±0.27%, respectively) emulsifier was achieved using 5 mg drug amounts (Fig. 6B and Fig. 6C, respectively). These results indicate an important role for drug solubility on the characterization of CEL loaded NPs.

CEL exhibits poor aqueous solubility and is categorized as a class II drug under the biopharmaceutical classification system [59]–[61]. During CEL loaded NP formulation, several techniques such as size reduction, use of emulsifier, or surfactants can be applied to help increase the degree of drug solubility in aqueous media and improve overall NP characteristics [62]. In applications oriented toward NP production, several of these techniques are often applied in order to increase drug solubility and prevent drug precipitating out of the NP shell. In this study, stabilizers and an emulsifier were used to alter drug solubility and optimize particle characteristics. The use of drug amount was also analyzed as a measurement of solubility effects on zeta potential, particle size, and drug entrapment. In an effort to optimize drug entrapment, drug amounts were titrated to measure extent of effects on NP encapsulation. The solubility of a drug is related to the ratio of drug surface area to solvent volume [62]. In particle size reduction, surface area is increased and allows greater interactions with the solvent which causes an increase in solubility. In conjunction with particle size reduction via sonication, reduction in drug amount improves drug solubility by further enhancing the surface-area-to-volume ratio. We found that when drug amount was decreased in CEL-NP formulations, entrapment efficiency was able to achieve over 95% loading capacitance (Fig. 6C). This success indicates the importance of drug amount in conjunction with size reduction for the prevention of drug precipitation and enhancement of entrapment efficiency during NP formulations.

In this study, we found that as drug amount increased to 20 mg, total entrapment of CEL was higher in formulations with emulsifier while amounts of 15 mg and 10 mg CEL displayed increasing total drug entrapment in regard to the emulsifier free formulation (Fig. 6C). It is possible that higher concentrations of CEL undergo enhanced solubilization in the presence of emulsifier enabling a larger degree of drug entrapment in the presence of higher drug amounts. Furthermore, lecithin is a non-ionic emulsifier known to impart steric stabilizing effects in colloidal systems, preventing particle collision and reducing drug leakage [63], [64]. It is possible that as drug amounts increase, lecithin functions to increase drug solubility, stabilize NP formation, and reduce drug leakage leading to an increase in drug entrapment. The observation that larger drug amounts undergo increased NP entrapment in the presence of lecithin may support the idea of emulsifier use during NP production of high concentrations of lipophilic drugs.

### Stability of CEL loaded PLGA-NPs

To avoid particle aggregation and coalescence, the recommended storage temperature for PLGA-NP systems is 4°C [65]. Therefore, to analyze the stability of PLGA-NP systems, emulsions of varying drug amounts with or without emulsifier were kept at 4°C for a period of 16 weeks then characterized to determine storage effects on zeta potential, particle size, and drug entrapment efficiency.

**Figure 7:**
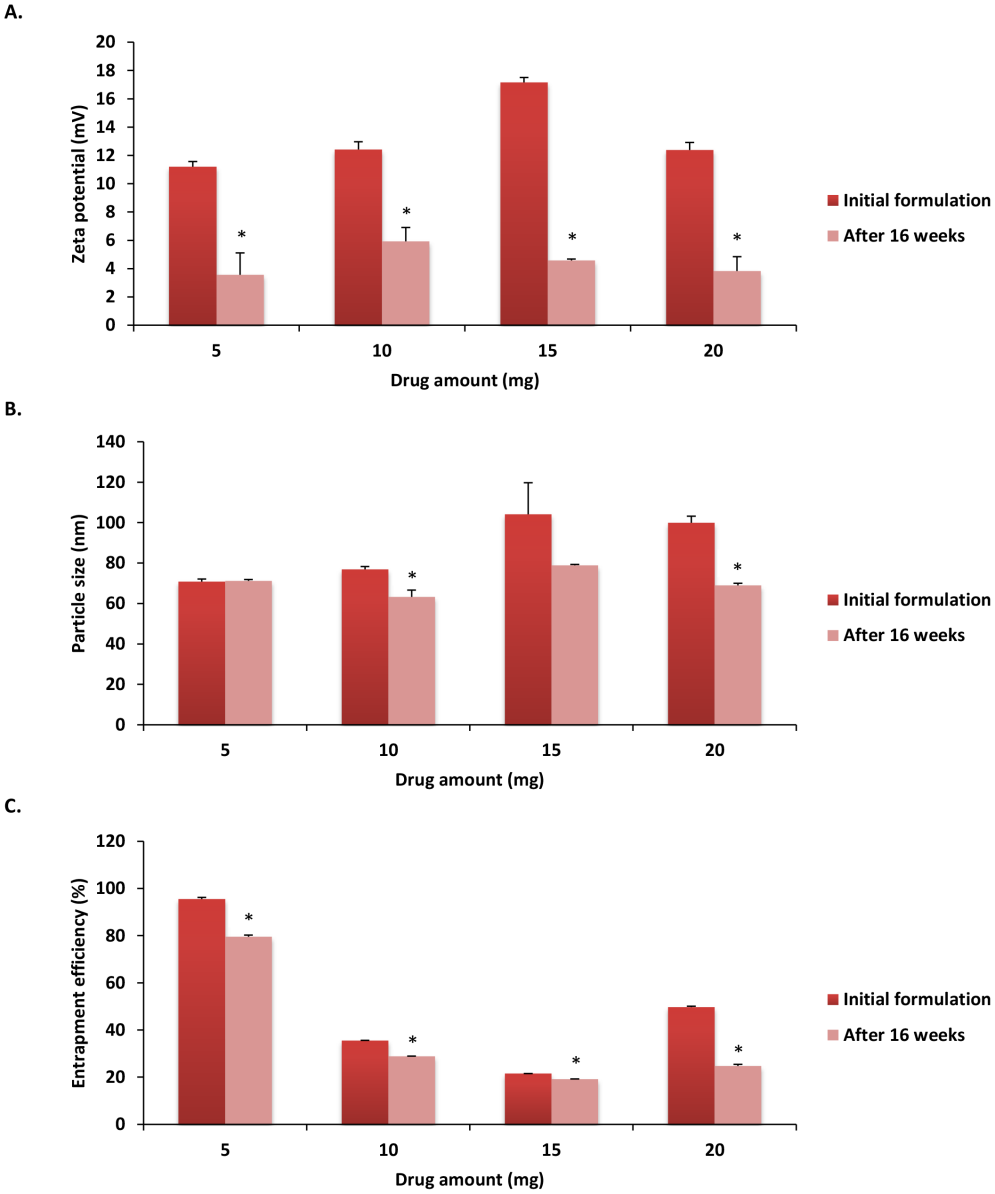
Nanoparticle characteristic comparison of A) zeta potential, B) particle size, and C) entrapment efficiency of initial emulsifier based formulations with those observed following 16 weeks cold storage at 4°C. Values are expressed as mean ± SD, n = 3. ^*^p<0.05, significantly different from initial formulations.

**Figure 8:**
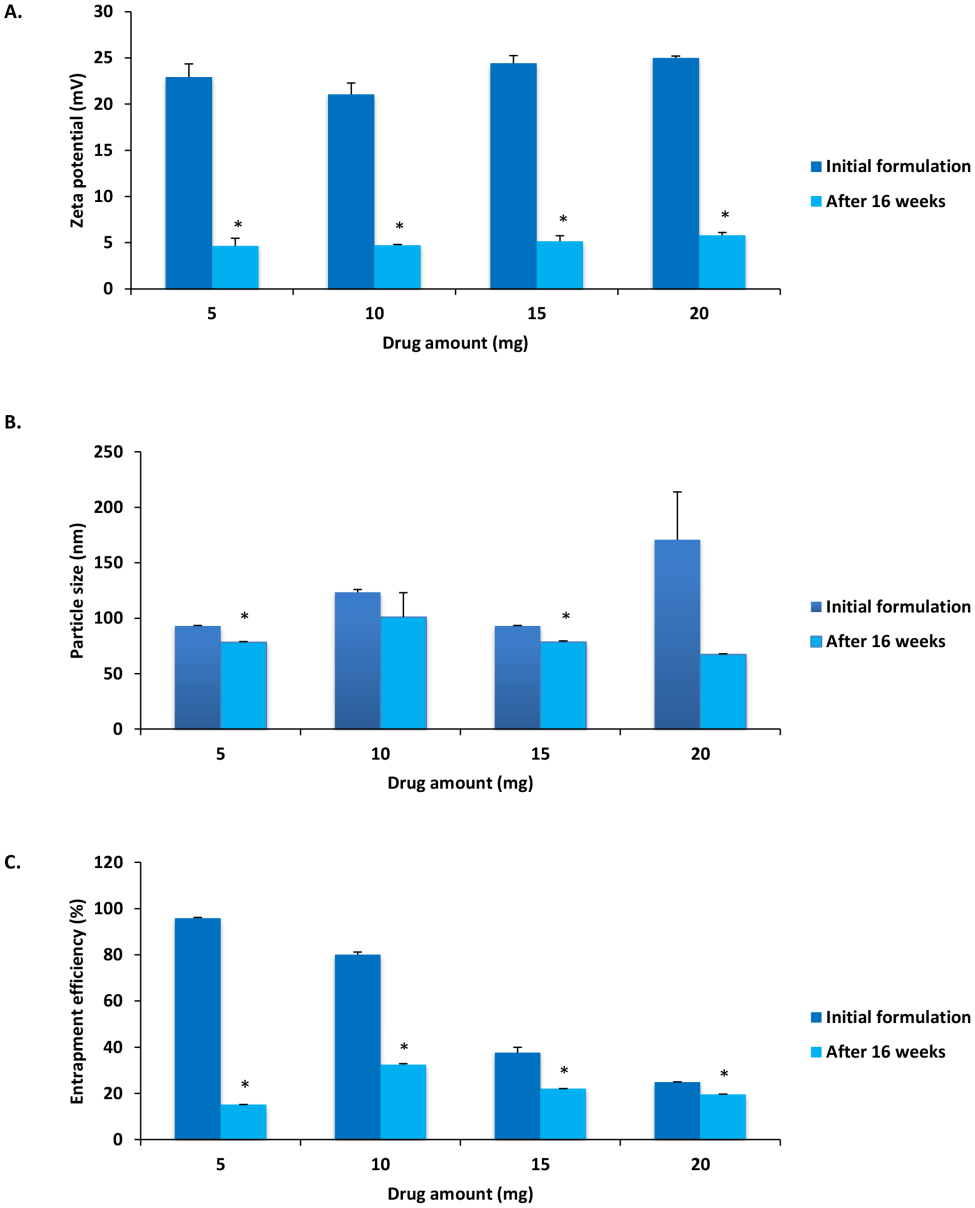
Nanoparticle characteristic comparison of A) zeta potential, B) particle size, and C) entrapment efficiency of initial emulsifier free formulations with those observed following 16 weeks cold storage at 4°C. Values are expressed as mean ± SD, n = 3. ^*^p<0.05, significantly different from initial formulations.

Results showed that zeta potential, particle size, and entrapment efficiency were at or below initial reported NP characterization measurements (Fig. 7 and Fig. 8). All peak characteristic measurements after 16 weeks of cold storage were noted in formulations that included emulsifier (Fig. 7). When compared to our initial formulations (Fig. 6), zeta potential was reduced across all formulations (p<0.05), with a peak zeta potential seen in formulations using 10 mg drug amounts with emulsifier (5.92±0.98 mV) (Fig. 7A). When analyzing particle diameter, a peak reduction was seen in 10 mg formulations with emulsifier (63.23±3.33 nm). Furthermore, when compared to initial characteristic measurements, significant particle size reduction was seen in the 10 mg and 20 mg CEL formulations with emulsifier (Fig. 7B) (p<0.01), as well as the 5 mg and 15 mg formulations without emulsifier (Fig. 8B) (p<0.01). All formulations showed a significant reduction (p<0.01) in entrapment efficiency with the highest level of entrapment maintained in the 5 mg formulation with emulsifier (79.58±0.611%) (Fig. 7C). These results indicate the possible role emulsifying agents may have in maintenance of NP stability. The reduction of zeta potential observed in all formulations could be a result of possible DMAB dissociation from the NP shell after 16 weeks. Loss of DMAB would lead to reduced particle charge, net repulsion, and stability resulting in increased drug leakage, particle size reduction, and reduced entrapment efficiency [66]. Furthermore, the emulsifier in our formulation may be exerting unknown effects on drug permeation and NP aggregation, allowing for enhanced time-dependent stability of PLGA-NPs formulated with lecithin [67].

## Conclusion

In this study, we performed a solvent evaporation technique to developed and characterize CEL loaded PLGA-NPs using varying concentrations of DMAB or PVA as stabilizer. NPs were examined and characterized based on zeta potential, size, drug entrapment efficiency, and morphology. The results of this study showed that the use of DMAB as stabilizer led to the development of NPs that displayed sufficient size and stability with moderate increases in drug entrapment when compared to plain formulation. Of the two stabilizers, DMAB proved to be highly efficient in developing well characterized CEL loaded PLGA based NPs, whereas PVA based formulations failed to reach optimum parameters in NP development. Variables such as emulsifier and drug amount were also analyzed to further optimize NP formulations. When formulations were carried out in the presence of emulsifier, a reduction in zeta potential was noted. Emulsifier based formulations displayed reduced surface charge as a consequence of lecithin induced anionic interactions and masking of cationic DMAB properties indicating that in the presence of DMAB based formulations emulsifiers such as lecithin may act to reduce NP stability and formula optimization. Additional formula evaluation showed that reduction in drug amount was effective at reducing particle size and enhancing drug entrapment efficiency further elucidating the role of drug solubility and the importance of increasing the surface-area-to-volume ratio for effective development of CEL loaded NPs. Interestingly, while the use of emulsifier resulted in reduced zeta potential and system stability, time-dependent stability testing, which looked at zeta potential, size, and entrapment efficiency after 16 weeks cold storage, showed peak particle characteristics in formulations with emulsifier. These results may indicate that while emulsifiers, such as lecithin, reduce overall particle charge during formulation, they could also prolong NP system stability over an extended period of time. However, further testing is needed to determine the extent of emulsifier effects on CEL loaded PLGA-NP stability. Overall, the results of our study indicate that the formulation of PLGA-NPs using 0.25% w/v DMAB and 5 mg CEL without emulsifier creates highly entrapped and stable NPs of a sufficient size that could function to enhance the application of orally delivered CEL and provide a potential effective dosage form for CEL administration.

## Supporting Information

S1 Figure TEM images of emulsifier based formulation illustrating morphology of A) 0.1% w/v DMAB formulated NPs, B) 0.25% w/v DMAB formulated NPs, C) 0.5% w/v DMAB formulated NPs, and D) 1% w/v DMAB formulated NPs.
(TIF)

S2 Figure TEM images of emulsifier free formulations illustrating morphology of A) 0.1% w/v DMAB formulated NPs, B) 0.25% w/v DMAB formulated NPs, C) 0.5% w/v DMAB formulated NPs, and D) 1% w/v DMAB formulated NPs.
(TIF)

## References

[1] Simmons DL, Botting RM, Hla T (2004) Cyclooxygenase isozymes: the biology of prostaglandin synthesis and inhibition. Pharmacol Rev 56:387–437. 15317910 10.1124/pr.56.3.3

[2] Fitzpatrick FA (2004) Cyclooxygenase enzymes: regulation and function. Curr Pharm Des 10:577–588. 14965321 10.2174/1381612043453144

[3] Seibert K, Masferrer JL (1994) Role of inducible cyclooxygenase (COX-2) in inflammation. Receptor 4:17–23. 8038702

[4] Seibert K, Masferrer J, Zhang Y, Gregory S, Olson G, et al**.** (1995) Mediation of inflammation by cyclooxygenase-2. Agents Actions Suppl 46 41–50. 10.1007/978-3-0348-7276-8_5 7610990

[5] Bertolini A, Ottani A, Sandrini M (2002) Selective COX-2 inhibitors and dual acting anti-inflammatory drugs: critical remarks. Curr Med Chem 9:1033–1043. 12733982 10.2174/0929867024606650

[6] Crofford LJ (1997) COX-1 and COX-2 tissue expression: implications and predictions. J Rheumatol Suppl 49 15–19. 9249646

[7] Seibert K, Zhang Y, Leahy K, Hauser S, Masferrer J, et al (1997) Distribution of COX-1 and COX-2 in normal and inflamed tissues. Adv Exp Med Biol 400A:167–170. 9547553 10.1007/978-1-4615-5325-0_24

[8] Zidar N, Odar K, Glavac D, Jerse M, Zupanc T, et al (2009) Cyclooxygenase in normal human tissues—is COX-1 really a constitutive isoform, and COX-2 an inducible isoform? J Cell Mol Med 13:3753–3763. 18657230 10.1111/j.1582-4934.2008.00430.x PMC4516524

[9] Hoffmann U, Banas B, Kruger B, Pietrzyk M, Obed A, et al (2006) Expression of cyclooxygenase-1 and cyclooxygenase-2 in human renal allograft rejection— a prospective study. Transpl Int 19:203–212. 16441769 10.1111/j.1432-2277.2005.00261.x

[10] Chen YF, Jobanputra P, Barton P, Bryan S, Fry-Smith A, et al (2008) Cyclooxygenase-2 selective non-steroidal anti-inflammatory drugs (etodolac, meloxicam, celecoxib, rofecoxib, etoricoxib, valdecoxib and lumiracoxib) for osteoarthritis and rheumatoid arthritis: a systematic review and economic evaluation. Health Technol Assess 12:1–278 iii. 10.3310/hta12110 18405470

[11] Clemett D, Goa KL (2000) Celecoxib: a review of its use in osteoarthritis, rheumatoid arthritis and acute pain. Drugs 59:957–980. 10804043 10.2165/00003495-200059040-00017

[12] Frampton JE, Keating GM (2007) Celecoxib: a review of its use in the management of arthritis and acute pain. Drugs 67:2433–2472. 17983259 10.2165/00003495-200767160-00008

[13] Harirforoosh S, Asghar W, Jamali F (2013) Adverse effects of nonsteroidal antiinflammatory drugs: an update of gastrointestinal, cardiovascular and renal complications. J Pharm Pharm Sci 16:821–847. 24393558 10.18433/j3vw2f

[14] McCormack PL (2011) Celecoxib: a review of its use for symptomatic relief in the treatment of osteoarthritis, rheumatoid arthritis and ankylosing spondylitis. Drugs 71:2457–2489. 22141388 10.2165/11208240-000000000-00000

[15] Chan FK, Hung LC, Suen BY, Wong VW, Hui AJ, et al (2004) Celecoxib versus diclofenac plus omeprazole in high-risk arthritis patients: results of a randomized double-blind trial. Gastroenterology 127:1038–1043. 15480981 10.1053/j.gastro.2004.07.010

[16] Goldenberg MM (1999) Celecoxib, a selective cyclooxygenase-2 inhibitor for the treatment of rheumatoid arthritis and osteoarthritis. Clin Ther 21:1497–1513 discussion 1427–1498. 10509845 10.1016/s0149-2918(00)80005-3

[17] Caldwell B, Aldington S, Weatherall M, Shirtcliffe P, Beasley R (2006) Risk of cardiovascular events and celecoxib: a systematic review and meta-analysis. J R Soc Med 99:132–140. 16508052 10.1258/jrsm.99.3.132 PMC1383759

[18] Ahmad SR, Kortepeter C, Brinker A, Chen M, Beitz J (2002) Renal failure associated with the use of celecoxib and rofecoxib. Drug Saf 25:537–544. 12093311 10.2165/00002018-200225070-00007

[19] Amrite AC, Ayalasomayajula SP, Cheruvu NP, Kompella UB (2006) Single periocular injection of celecoxib-PLGA microparticles inhibits diabetes-induced elevations in retinal PGE2, VEGF, and vascular leakage. Invest Ophthalmol Vis Sci 47:1149–1160. 16505053 10.1167/iovs.05-0531 PMC3324981

[20] Ayalasomayajula SP, Kompella UB (2005) Subconjunctivally administered celecoxib-PLGA microparticles sustain retinal drug levels and alleviate diabetes-induced oxidative stress in a rat model. Eur J Pharmacol 511:191–198. 15792788 10.1016/j.ejphar.2005.02.019

[21] Bachar M, Mandelbaum A, Portnaya I, Perlstein H, Even-Chen S, et al (2012) Development and characterization of a novel drug nanocarrier for oral delivery, based on self-assembled beta-casein micelles. J Control Release 160:164–171. 22266050 10.1016/j.jconrel.2012.01.004

[22] Dhanda DS, Tyagi P, Mirvish SS, Kompella UB (2013) Supercritical fluid technology based large porous celecoxib-PLGA microparticles do not induce pulmonary fibrosis and sustain drug delivery and efficacy for several weeks following a single dose. J Control Release 168:239–250. 23562638 10.1016/j.jconrel.2013.03.027

[23] Morgen M, Bloom C, Beyerinck R, Bello A, Song W, et al (2012) Polymeric nanoparticles for increased oral bioavailability and rapid absorption using celecoxib as a model of a low-solubility, high-permeability drug. Pharm Res 29:427–440. 21863477 10.1007/s11095-011-0558-7 PMC3264876

[24] Nguyen TH, Tan A, Santos L, Ngo D, Edwards GA, et al (2013) Silica-lipid hybrid (SLH) formulations enhance the oral bioavailability and efficacy of celecoxib: An in vivo evaluation. J Control Release 167:85–91. 23353808 10.1016/j.jconrel.2013.01.012

[25] Tan A, Davey AK, Prestidge CA (2011) Silica-lipid hybrid (SLH) versus non-lipid formulations for optimising the dose-dependent oral absorption of celecoxib. Pharm Res 28:2273–2287. 21560021 10.1007/s11095-011-0458-x

[26] Thakkar H, Kumar Sharma R, Murthy RS (2007) Enhanced retention of celecoxib-loaded solid lipid nanoparticles after intra-articular administration. Drugs R D 8:275–285. 17767393 10.2165/00126839-200708050-00002

[27] Shakeel F, Baboota S, Ahuja A, Ali J, Shafiq S (2009) Enhanced anti-inflammatory effects of celecoxib from a transdermally applied nanoemulsion. Pharmazie 64:258–259. 19435145

[28] Venkatesan P, Puvvada N, Dash R, Prashanth Kumar BN, Sarkar D, et al (2011) The potential of celecoxib-loaded hydroxyapatite-chitosan nanocomposite for the treatment of colon cancer. Biomaterials 32:3794–3806. 21392822 10.1016/j.biomaterials.2011.01.027

[29] Parhi P, Mohanty C, Sahoo SK (2012) Nanotechnology-based combinational drug delivery: an emerging approach for cancer therapy. Drug Discov Today 17:1044–1052. 22652342 10.1016/j.drudis.2012.05.010

[30] Danhier F, Ansorena E, Silva JM, Coco R, Le Breton A, et al (2012) PLGA-based nanoparticles: an overview of biomedical applications. J Control Release 161:505–522. 22353619 10.1016/j.jconrel.2012.01.043

[31] Rao DA, Forrest ML, Alani AW, Kwon GS, Robinson JR (2010) Biodegradable PLGA based nanoparticles for sustained regional lymphatic drug delivery. J Pharm Sci 99:2018–2031. 19902520 10.1002/jps.21970 PMC5178132

[32] Vasir JK, Labhasetwar V (2007) Biodegradable nanoparticles for cytosolic delivery of therapeutics. Adv Drug Deliv Rev 59:718–728. 17683826 10.1016/j.addr.2007.06.003 PMC2002520

[33] Cooper DL, Harirforoosh S (2014) Design and optimization of PLGA-based diclofenac loaded nanoparticles. PLoS One 9:e87326. 24489896 10.1371/journal.pone.0087326 PMC3905017

[34] Sahana DK, Mittal G, Bhardwaj V, Kumar MN (2008) PLGA nanoparticles for oral delivery of hydrophobic drugs: influence of organic solvent on nanoparticle formation and release behavior in vitro and in vivo using estradiol as a model drug. J Pharm Sci 97:1530–1542. 17722098 10.1002/jps.21158

[35] Italia JL, Bhatt DK, Bhardwaj V, Tikoo K, Kumar MN (2007) PLGA nanoparticles for oral delivery of cyclosporine: nephrotoxicity and pharmacokinetic studies in comparison to Sandimmune Neoral. J Control Release 119:197–206. 17399839 10.1016/j.jconrel.2007.02.004

[36] Bhardwaj V, Ankola DD, Gupta SC, Schneider M, Lehr CM, et al (2009) PLGA nanoparticles stabilized with cationic surfactant: safety studies and application in oral delivery of paclitaxel to treat chemical-induced breast cancer in rat. Pharm Res 26:2495–2503. 19756974 10.1007/s11095-009-9965-4

[37] Qiao JL, Zhang J, Zhang JJ (2013) Anion conducting poly(vinyl alcohol)/poly(diallyldimethylammonium chloride) membranes with high durable alkaline stability for polymer electrolyte membrane fuel cells. Journal of Power Sources 237:1–4.

[38] Hariharan S, Bhardwaj V, Bala I, Sitterberg J, Bakowsky U, et al (2006) Design of estradiol loaded PLGA nanoparticulate formulations: a potential oral delivery system for hormone therapy. Pharm Res 23:184–195. 16267632 10.1007/s11095-005-8418-y

[39] Labhasetwar V, Song C, Humphrey W, Shebuski R, Levy RJ (1998) Arterial uptake of biodegradable nanoparticles: effect of surface modifications. J Pharm Sci 87:1229–1234. 9758682 10.1021/js980021f

[40] Immordino ML, Dosio F, Cattel L (2006) Stealth liposomes: review of the basic science, rationale, and clinical applications, existing and potential. Int J Nanomedicine 1:297–315. 17717971 PMC2426795

[41] Gomaa YA, Garland MJ, McInnes FJ, Donnelly RF, El-Khordagui LK, et al (2014) Microneedle/nanoencapsulation-mediated transdermal delivery: mechanistic insights. Eur J Pharm Biopharm 86:145–155. 23461860 10.1016/j.ejpb.2013.01.026 PMC4074889

[42] Bala I, Bhardwaj V, Hariharan S, Kharade SV, Roy N, et al (2006) Sustained release nanoparticulate formulation containing antioxidant-ellagic acid as potential prophylaxis system for oral administration. J Drug Target 14:27–34. 16603449 10.1080/10611860600565987

[43] Xu A, Yao M, Xu G, Ying J, Ma W, et al (2012) A physical model for the size-dependent cellular uptake of nanoparticles modified with cationic surfactants. Int J Nanomedicine 7:3547–3554. 22848178 10.2147/IJN.S32188 PMC3405883

[44] Mosqueira VC, Legrand P, Pinto-Alphandary H, Puisieux F, Barratt G (2000) Poly (D, L-lactide) nanocapsules prepared by a solvent displacement process: influence of the composition on physicochemical and structural properties. J Pharm Sci 89:614–626. 10756327 10.1002/(SICI)1520-6017(200005)89:5<614::AID-JPS7>3.0.CO;2-7

[45] Schubert MA, Muller-Goymann CC (2005) Characterisation of surface-modified solid lipid nanoparticles (SLN): influence of lecithin and nonionic emulsifier. Eur J Pharm Biopharm 61:77–86. 16011893 10.1016/j.ejpb.2005.03.006

[46] Fisk ID, Linforth R, Trophardy G, Gray D (2013) Entrapment of a volatile lipophilic aroma compound (d-limonene) in spray dried water-washed oil bodies naturally derived from sunflower seeds (). Food Res Int 54:861–866. 24235784 10.1016/j.foodres.2013.08.024 PMC3824067

[47] Hills BA (1983) Contact-angle hysteresis induced by pulmonary surfactants. J Appl Physiol Respir Environ Exerc Physiol 54:420–426. 6687588 10.1152/jappl.1983.54.2.420

[48] Rodriguez M, Oses J, Ziani K, Mate JI (2006) Combined effect of plasticizers and surfactants on the physical properties of starch based edible films. Food Research International 39:840–846.

[49] Yamamoto Y, Araki M (1997) Effects of lecithin addition in oil or water phase on the stability of emulsions made with whey proteins. Biosci Biotechnol Biochem 61:1791–1795. 9404055 10.1271/bbb.61.1791

[50] Thakkar H, Sharma RK, Mishra AK, Chuttani K, Murthy RR (2005) Albumin microspheres as carriers for the antiarthritic drug celecoxib. AAPS PharmSciTech 6:E65–73. 16353965 10.1208/pt060112 PMC2750413

[51] Chen JB, Gao HW, Zhang YL, Zhang Y, Zhou XF, et al (2014) Developmental toxicity of diclofenac and elucidation of gene regulation in zebrafish (Danio rerio). Sci Rep 4:4841. 24788080 10.1038/srep04841 PMC4007093

[52] Amrite A, Pugazhenthi V, Cheruvu N, Kompella U (2010) Delivery of celecoxib for treating diseases of the eye: influence of pigment and diabetes. Expert Opin Drug Deliv 7:631–645. 20205602 10.1517/17425241003663236 PMC2858240

[53] Kenawi IM, Kamel AH, Hilal RH (2008) BSSE effects on the static dipole polarizability and first dipole hyperpolarizability of diclofenac sodium. Journal of Molecular Structure: THEOCHEM 851:46–53.

[54] Joshi M, Patravale V (2008) Nanostructured lipid carrier (NLC) based gel of celecoxib. Int J Pharm 346:124–132. 17651933 10.1016/j.ijpharm.2007.05.060

[55] Tan A, Simovic S, Davey AK, Rades T, Prestidge CA (2009) Silica-lipid hybrid (SLH) microcapsules: a novel oral delivery system for poorly soluble drugs. J Control Release 134:62–70. 19013488 10.1016/j.jconrel.2008.10.014

[56] Palamoor M, Jablonski MM (2013) Synthesis, characterization and in vitro studies of celecoxib-loaded poly(ortho ester) nanoparticles targeted for intraocular drug delivery. Colloids Surf B Biointerfaces 112:474–482. 24103464 10.1016/j.colsurfb.2013.07.039

[57] Govender T, Stolnik S, Garnett MC, Illum L, Davis SS (1999) PLGA nanoparticles prepared by nanoprecipitation: drug loading and release studies of a water soluble drug. J Control Release 57:171–185. 9971898 10.1016/s0168-3659(98)00116-3

[58] Song X, Zhao Y, Hou S, Xu F, Zhao R, et al (2008) Dual agents loaded PLGA nanoparticles: systematic study of particle size and drug entrapment efficiency. Eur J Pharm Biopharm 69:445–453. 18374554 10.1016/j.ejpb.2008.01.013

[59] Lakshmi K, Reddy MP, Kaza R (2013) Design and characterization of microcrystals for enhanced dissolution rate of celecoxib. Curr Drug Discov Technol 10:305–314. 24074306 10.2174/15701638113109990035

[60] Xu Y, Mao L, Li X, Wang Y, Wei P (2013) Dissolution improvement of poorly water-soluble drug by cogrinding method using jar mill. Pak J Pharm Sci 26:495–502. 23625422

[61] Han C, Zhang J, Zheng M, Xiao Y, Li Y, et al (2011) An integrated drug-likeness study for bicyclic privileged structures: from physicochemical properties to in vitro ADME properties. Mol Divers 15:857–876. 21538133 10.1007/s11030-011-9317-2

[62] Savjani KT, Gajjar AK, Savjani JK (2012) Drug solubility: importance and enhancement techniques. ISRN Pharm 2012:195727. 22830056 10.5402/2012/195727 PMC3399483

[63] Zainol S, Basri M, Basri HB, Shamsuddin AF, Abdul-Gani SS, et al (2012) Formulation optimization of a palm-based nanoemulsion system containing levodopa. Int J Mol Sci 13:13049–13064. 23202937 10.3390/ijms131013049 PMC3497311

[64] Crosasso P, Ceruti M, Brusa P, Arpicco S, Dosio F, et al (2000) Preparation, characterization and properties of sterically stabilized paclitaxel-containing liposomes. J Control Release 63:19–30. 10640577 10.1016/s0168-3659(99)00166-2

[65] De S, Robinson DH (2004) Particle size and temperature effect on the physical stability of PLGA nanospheres and microspheres containing Bodipy. AAPS PharmSciTech 5:e53. 15760050 10.1208/pt050453 PMC2750478

[66] Mohammadi Z, Dorkoosh FA, Hosseinkhani S, Amini T, Rahimi AA, et al (2012) Stability studies of chitosan-DNA-FAP-B nanoparticles for gene delivery to lung epithelial cells. Acta Pharm 62:83–92. 22472451 10.2478/v10007-012-0008-z

[67] Hu K, Cao S, Hu F, Feng J (2012) Enhanced oral bioavailability of docetaxel by lecithin nanoparticles: preparation, in vitro, and in vivo evaluation. Int J Nanomedicine 7:3537–3545. 22848177 10.2147/IJN.S32880 PMC3405895

