# Supplementary figures and images for: Effect of Formulation Variables on Preparation of Celecoxib Loaded Polylactide-Co-Glycolide Nanoparticles
Source: PLoS One. 2014 Dec 12;9(12):e113558. doi: 10.1371/journal.pone.0113558 (PMC4264745; doi:10.1371/journal.pone.0113558)

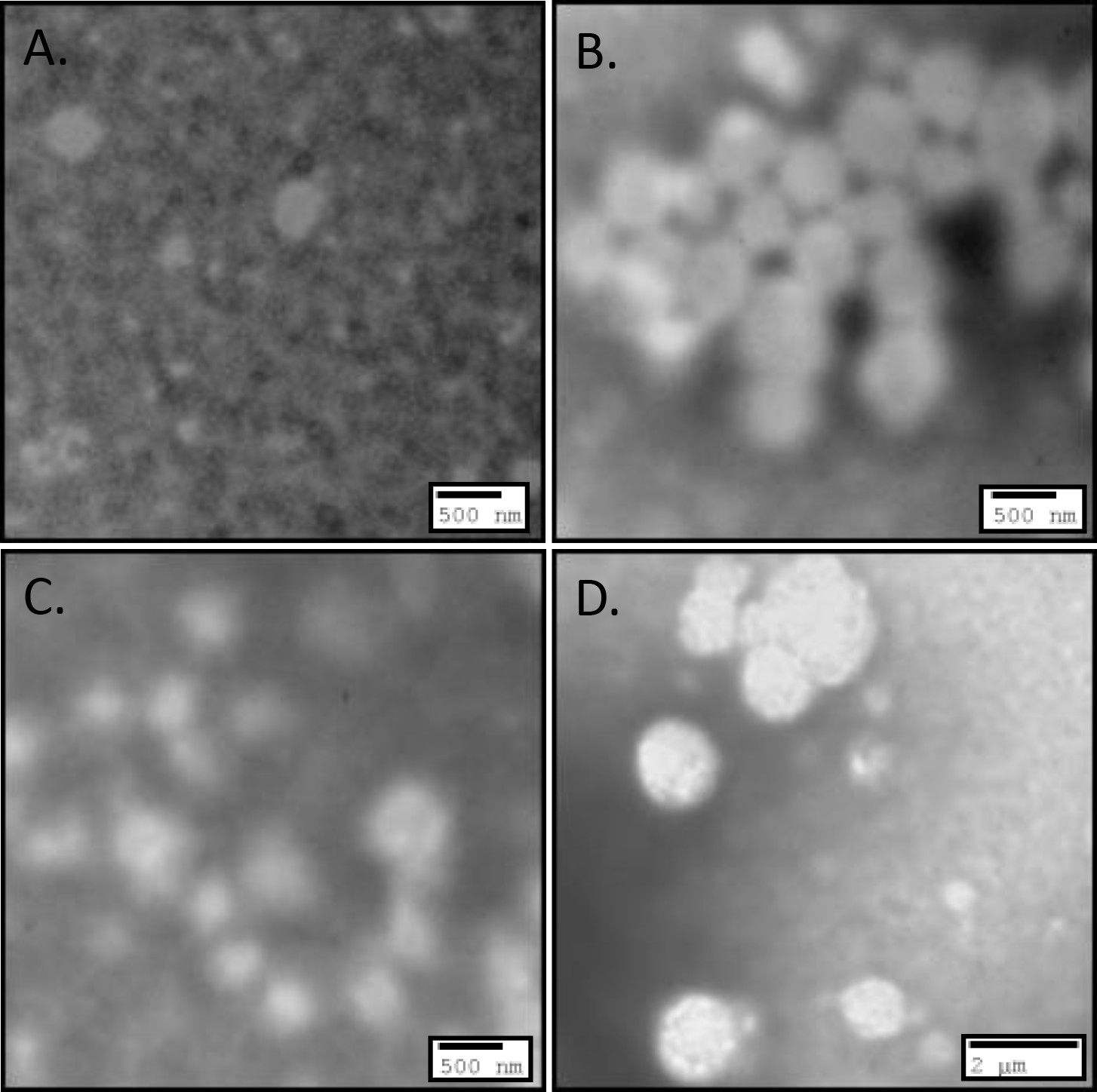

Supplement: S1 Figure — TEM images of emulsifier based formulation illustrating morphology of A) 0.1% w/v DMAB formulated NPs, B) 0.25% w/v DMAB formulated NPs, C) 0.5% w/v DMAB formulated NPs, and D) 1% w/v DMAB formulated NPs. (TIF) [file pone.0113558.s001.tif]

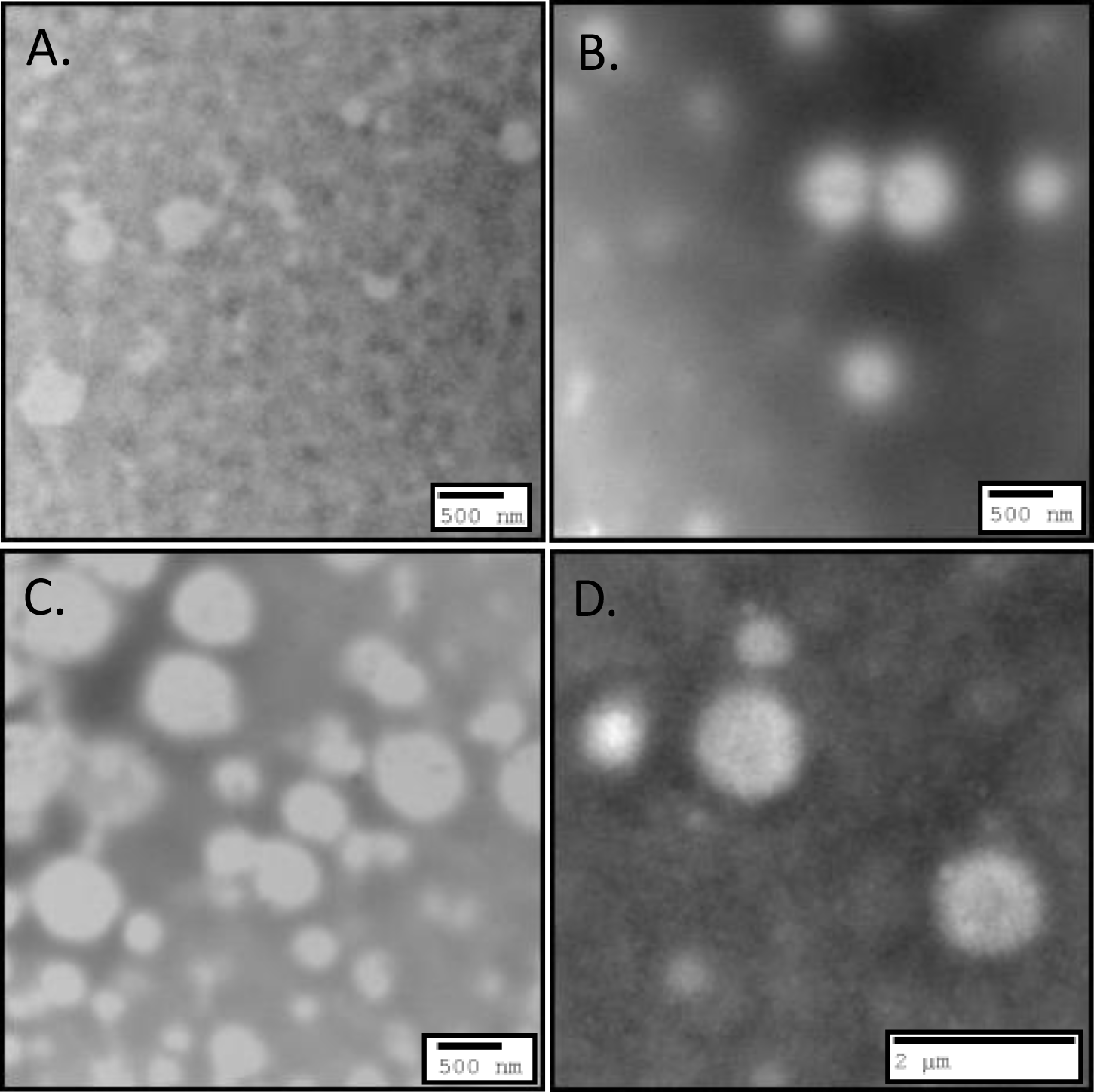

Supplement: S2 Figure — TEM images of emulsifier free formulations illustrating morphology of A) 0.1% w/v DMAB formulated NPs, B) 0.25% w/v DMAB formulated NPs, C) 0.5% w/v DMAB formulated NPs, and D) 1% w/v DMAB formulated NPs. (TIF) [file pone.0113558.s002.tif]
